# The epidemiology of gastrointestinal stromal tumors in Taiwan, 1998–2008: a nation-wide cancer registry-based study

**DOI:** 10.1186/1471-2407-14-102

**Published:** 2014-02-18

**Authors:** Nai-Jung Chiang, Li-Tzong Chen, Chia-Rung Tsai, Jeffrey S Chang

**Affiliations:** 1 National Institute of Cancer Research, National Health Research Institutes, 2 F, No. 367, Sheng Li Road, Tainan 70456, Taiwan; 2 Division of Hematology/Oncology, Department of Internal Medicine, National Cheng Kung University Hospital, 138 Sheng Li Road, Tainan 70456, Taiwan; 3 Department of Internal Medicine, Kaohsiung Medical University Hospital, Kaohsiung Medical University, No. 100, Ziyou 1st. Road, Sanmin District, Kaohsiung 807, Taiwan; 4 School of Pharmacy, College of Pharmacy, Taipei Medical University, 250 Wu-Hsing Street, Taipei 110, Taiwan

**Keywords:** Gastrointestinal stromal tumors, Incidence, Imatinib, Survival

## Abstract

**Background:**

To investigate the incidence of gastrointestinal stromal tumors (GISTs) in Taiwan and the impact of imatinib on the overall survival (OS) of GIST patients.

**Methods:**

GISTs were identified from the Taiwan Cancer Registry (TCR) from 1998 to 2008. The age-adjusted incidence rates and the observed OS rates were calculated. Cox proportional hazards models were applied to examine the mortality risk in three time periods (1998–2001, 2002–2004, 2005–2008) according to the application and availability of imatinib.

**Results:**

From 1998 to 2008, 2,986 GISTs were diagnosed in Taiwan. The incidence increased from 1.13 per 100,000 in 1998 to 1.97 per 100,000 in 2008. The most common sites were stomach (47-59%), small intestine (31-38%), and colon/rectum (6-9%). The 5-year observed OS was 66.5% (60.3% for men, 74.2% for women, *P* < .0001). GISTs in the stomach had a better 5-year observed OS (69.4%) than those in the small intestine (65.1%) (*P* < .0001). The outcome of GIST improved significantly after the more widespread use of imatinib; the 5-year observed OS increased from 58.9% during 1998–2001 to 70.2% during 2005–2008 (*P* < .0001). Younger age, female sex, stomach location, and later diagnostic years were independent predictors of a better survival.

**Conclusions:**

The incidence of GIST has been increasing in Taiwan, partially due to the advancement of diagnostic technology/method and the increased awareness by physicians. The outcome of GIST has improved significantly with the availability and the wider use of imatinib.

## Background

Gastrointestinal stromal tumors (GISTs) are the most common mesenchymal neoplasms of the gastrointestinal system, characterized by an unique histological morphology and the expression of the KIT protein [1]. Previously, the majority of GISTs were diagnosed as smooth muscle tumors (e.g*.* leiomyoma and leiomyosarcoma) or as tumors of the nerve sheath origin (*e.g.* schwannoma and malignant nerve sheath tumors) [2,3]. Because GISTs were previously difficult to define due to the lack of specific markers, few epidemiologic studies were published with no nation-wide cancer registry-based study of GISTs from Asia [4,5]. The advancement of immunohistochemistry, molecular technology and the identification of *KIT* oncogene mutation in more than 80% of GISTs have accelerated our understanding of GISTs [6-8]. In Taiwan, the diagnosis of GISTs by CD117 or KIT staining was established and widely adopted since 2002. Prior to 2002, the diagnosis of GISTs was based on histology and other immunohistochemical markers (CD34, vimentin, keratin, smooth muscle actin (SMA), and S100) [4,9]. Using the Taiwan Cancer Registry (TCR) data from 1998 to 2008, our analysis elucidated the incidence and the distribution of GISTs before and after the implementation of CD117 or KIT staining for the definitive diagnosis of GISTs and compared them to those in the Western countries.

Complete surgical resection remains the only curative treatment of primary localized GISTs. The 5-yr survival rate after complete surgical resection was 50% before the era of molecular targeted therapy [10,11]. The approval of imatinib mesylate (Gleevec®, Novartis Pharma, Basel, Switzerland), an oral inhibitor of KIT and platelet-derived growth factor receptor, alpha polypeptide (PDGFRA), to treat metastatic GIST by the USA FDA in 2002 has markedly changed the outcomes and treatment options for GISTs [12]. In Taiwan, imatinib was approved for reimbursement by the National Health Insurance Administration since 2004. Our analysis assessed the survival of GISTs by three time periods: 1) 1998–2001, before the approval imatinib to treat GISTs; 2) 2002–2004, after the approval of imatinib to treat GISTs and before the coverage of imatinib by the National Health Insurance of Taiwan; and 3) 2005–2008, after the coverage of imatinib by the National Health Insurance of Taiwan.

## Methods

### Data sources

The GIST cases diagnosed from January 1, 1998 to December 31, 2008 were identified from the TCR established in 1979 to track the cancer incidence and mortality in Taiwan [13]. Hospitals with more than 50 beds in Taiwan are mandated to report confirmed cases of malignancy to the TCR, which captures 97% of the cancer cases in Taiwan [13]. The quality of a cancer registry is measured by the percentage of death certificate only cases (DCO%) and the percentage of morphologically verified cases (MV%), with a DCO% of 0 and a MV% of 100 representing a perfect data quality [14]. The quality of the TCR is comparable to the other well-established cancer registries in the world [15,16] with a DCO% of 1.2% and a MV% of 89% [13].

### Study population

Before 2002, the diagnosis of GISTs by CD117 or c-KIT staining was unavailable; therefore, for cases diagnosed from January 1, 1998 to December 31, 2001, the morphology (M) codes of the International Classification of Disease for Oncology, Field Trial Edition (ICD-O-FT) were used to identify GIST cases with the algorithm established by Tran *et al.*[17], which included stromal sarcoma (8930), leiomyosarcoma (8890), epithelioid leiomyosarcoma (8891), cellular leiomyosarcoma (8892), bizarre leiomyosarcoma (8893), myxoid leiomyosarcoma (8896), smooth muscle cell tumor (8897), sarcoma not otherwise specified (8800), spindle cell sarcoma (8801), giant cell sarcoma (8802), small cell sarcoma (8803), epithelioid sarcoma (8804), mesenchymoma (8990), fibrosarcoma (8810), fibromyxosarcoma (8811), ganglioneuroma (9490), ganglioneuromatosis (9491), neurobalstoma (9500), neuroepithelioma (9503), ganglioglioma (9505), neurofibroma (9504), schwannoma (9650), paragangmaluganglioma (8680), glomus tumor (8711), angiosarcoma (9120), and hemangiopericytoma (9150). The origin of tumors was limited to the following primary sites: esophagus, stomach, small intestine, colon and rectum. In addition, only those with confirmed malignant behavior by histological criteria (ICD-O-FT fifth digit of /3) were included.

GISTs diagnosed after January 1, 2002 were identified by the International classification of Diseases for Oncology, Third Edition (ICD-O-3) with the M code for gastrointestinal stromal sarcoma (8936). Only cases with confirmed malignant neoplasm (ICD-O-3 fifth digit of /3) were included.

### Statistical analysis

The crude annual incidence was calculated by dividing the number of annual incident GIST cases by the annual population reported by the Directorate-General of Budget, Accounting, and Statistics of Taiwan ( http://www.dgbas.gov.tw). The crude incidence rates were calculated for all GISTs combined, by sex, and by primary sites. All incidence rates (per 100,000) were age-adjusted to the 2000 U.S. standard population to generate the age-standardized incidence rates. The observed overall survival (OS) rates were calculated for all patients and by sex, primary sites, and diagnostic periods. Patients were followed from the date of diagnosis to death recorded in the national death database or to the end of follow-up on December 31, 2010. Cox proportional hazards models were performed to generate hazard ratios (HRs) and 95% confidence intervals (CIs) for the risk of mortality associated with tumor site, sex, age, and the year of diagnosis. Stage at diagnosis (localized or metastatic), tumor size, and mitotic index were excluded from the analysis because of incomplete or lack of information. This study was approved by the Institutional Review Board of the National Health Research Institutes.

## Results

### Characteristics of GIST patients

During 1998–2008, 2,986 newly diagnosed GIST cases were recorded by the TCR. The age of GIST patients ranged from 18 to 96 years old. The median age was around 62–64 years old and almost 75% of cases were diagnosed at ≧50 years of age (Table 1). For both sexes, the most common primary sites of GISTs were stomach (47-59%), followed by small intestine (31-38%), and colon/rectum (6-9%).

**Table 1:** Characteristics of gastrointestinal stromal tumors patients by three time periods, Taiwan, 1998-2008

**Age, years**	**Time period**					
		**1998-2001 N = 655**		**2002-2004 N = 846**		**2005-2008 N = 1485**	
	**Male N = 376**	**Female N = 279**	**Male N = 464**	**Female N = 382**	**Male N = 801**	**Female N = 684**	
<50	100 (26.6%)	78 (28.0%)	115 (24.8%)	86 (22.5%)	159 (19.9%)	129 (18.9%)
50 to <60	70 (18.6%)	50 (17.9%)	89 (19.2%)	91 (23.8%)	191 (23.9%)	162 (23.7%)
60 to <70	108 (27.1%)	74 (26.5%)	116 (25%)	96 (25.1%)	200 (25.0%)	184 (26.9%)
70 to <80	78 (20.7%)	68 (24.4%)	118 (25.4%)	86 (22.5%)	182 (22.7%)	157 (23.0%)
>80	26 (6.9%)	9 (3.2%)	26 (5.6%)	23 (6.0%)	69 (8.6%)	52 (7.6%)
Median (min/max)	62 (26/87)	62 (23/88)	63 (19/89)	62 (18/91)	64 (19/96)	64 (22/94)
**Location**						
Stomach	199 (52.9%)	164 (58.8%)	217 (46.8%)	196 (51.3%)	395 (49.3%)	375 (54.8%)
Small intestine	143 (38.0%)	94 (33.7%)	177 (38.2%)	125 (32.7%)	289 (36.1%)	213 (31.1%)
Colon/rectum	30 (8.0%)	20 (7.2%)	39 (8.4%)	34 (8.9%)	45 (5.6%)	43 (6.3%)
Esophagus/others^a^	4 (1.1%)	1 (0.4%)	31 (6.7%)	27 (7.1%)	72 (9.0%)	53 (7.8%)

^a^Others: retroperitoneum and unspecific sites.

A higher percentage of GIST originated from the small intestine was observed among those aged <50 years, and this percentage decreased with increasing age (from 43.8% among those younger than 50 years to 28.7% for those aged 70 years or older in 1998–2001; from 42.1% among those younger than 50 years to 31.4% for those aged 70 years or older in 2002–2008 (Table 2). In contrast, the percentage of GIST originated from the stomach increased with age (from 48.3% among those younger than 50 years to 63.0% for those aged 70 years or older in 1998–2001; from 42.9% among those younger than 50 years to 54.3% for those aged 70 years or older in 2002–2008). No significant trend was observed for the percentages of GISTs in colon/rectum or esophagus/others by increasing age.

**Table 2:** Distribution of gastrointestinal stromal tumors by age groups and locations, Taiwan, 1998–2008

**Location**	**1998-2001**			
	**Age (years)**			
	**<50 N = 178**	**50 to < 60 N = 120**	**60 to < 70 N = 176**	**≥ 70 N = 181**
Stomach	86 (48.3%)	68 (56.7%)	95 (54.0%)	114 (63.0%)
Small intestine	78 (43.8%)	41 (43.8%)	66 (37.5%)	52 (28.7%)
Colon/rectum	12 (6.7%)	11 (9.2%)	15 (8.5%)	12 (6.6%)
Esophagus/others^a^	2 (1.1%)	0 (0.0%)	0 (0.0%)	3 (1.7%)
	**2002-2008**			
**Location**	**<50 N = 489**	**50 to < 60 N = 533**	**60 to < 70 N = 596**	**≥ 70 N = 713**
Stomach	210 (42.9%)	266 (45.0%)	320 (53.7%)	387 (54.3%)
Small intestine	206 (42.1%)	182 (34.2%)	192 (32.2%)	224 (31.4%)
Colon/rectum	36 (7.4%)	45 (8.4%)	40 (6.7%)	40 (5.6%)
Esophagus/others^a^	37 (7.6%)	40 (7.5%)	44 (7.4%)	62 (8.7%)

^a^Others: retroperitoneum and unspecific sites.

### Incidence rates of GIST

**Figure 1:**
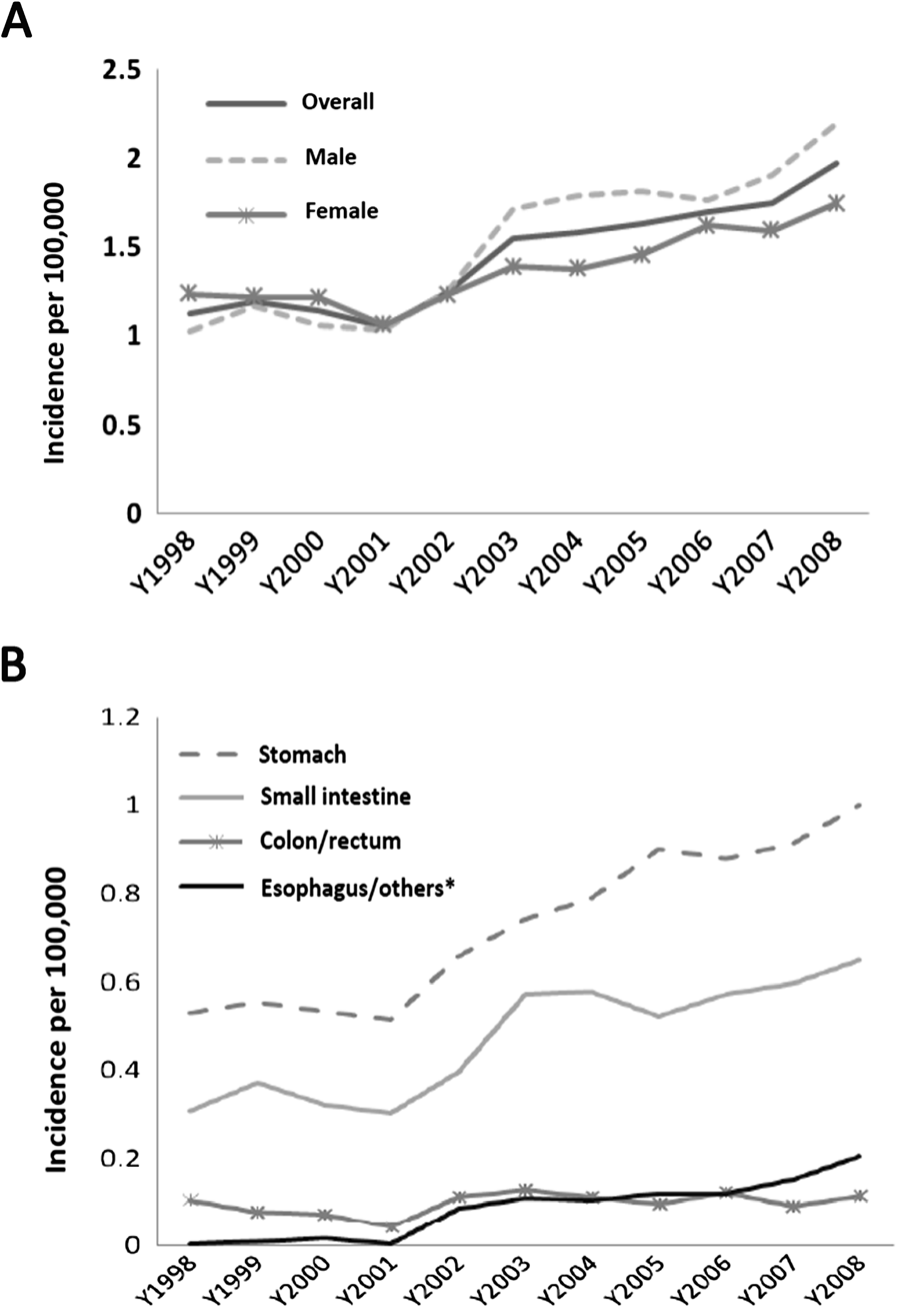
The incidence rate of gastrointestinal stromal tumors, Taiwan, 1998–2008: A) Overall and by sex; B) By primary sites.

The age-standardized annual incidence rate increased from 1.13/100,000 in 1998, to 1.25/100,000 in 2002, and to 1.97/100,000 in 2008 (Table 3 and Figure 1A). During 2002–2008, men consistently had a slightly higher incidence rate of GIST than women (male to female ratio ranged from 1.02 to 1.26). The incidence rate increased gradually during 1998–2008, but more prominently after 2002. There was a rise in the incidence of GIST located in stomach, small intestine, and esophagus/others(retroperitoneum and unspecified sites), whereas the incidence of GIST in colon/rectum remained relatively stable (Table 3 and Figure 1B).

**Table 3:** **Age-standardized incidence (per 100,000) of gastrointestinal stromal tumors, Taiwan, 1998-2008**^**a**^

**Year**	**Total**	**Gender**		**Primary sites**			
		**Male**	**Female**	**Stomach**	**Small intestine**	**Colon/rectum**	**Esophagus/others**^**b**^
1998	1.13	1.03	1.24	0.53	0.31	0.11	0.01
1999	1.20	1.17	1.22	0.56	0.37	0.08	0.01
2000	1.14	1.06	1.22	0.53	0.32	0.07	0.02
2001	1.05	1.04	1.07	0.52	0.30	0.04	0.00
2002	1.25	1.26	1.23	0.66	0.40	0.11	0.09
2003	1.55	1.72	1.39	0.74	0.57	0.13	0.11
2004	1.59	1.79	1.38	0.79	0.58	0.11	0.10
2005	1.64	1.81	1.46	0.9	0.52	0.10	0.12
2006	1.70	1.77	1.63	0.88	0.57	0.12	0.12
2007	1.75	1.91	1.59	0.91	0.60	0.09	0.15
2008	1.97	2.20	1.75	1.00	0.65	0.12	0.20

^a^Age-standardized to the 2000 US standard population.

^b^Others: retroperitoneum and unspecific sites.

### Survival of GIST patients

**Figure 2:**
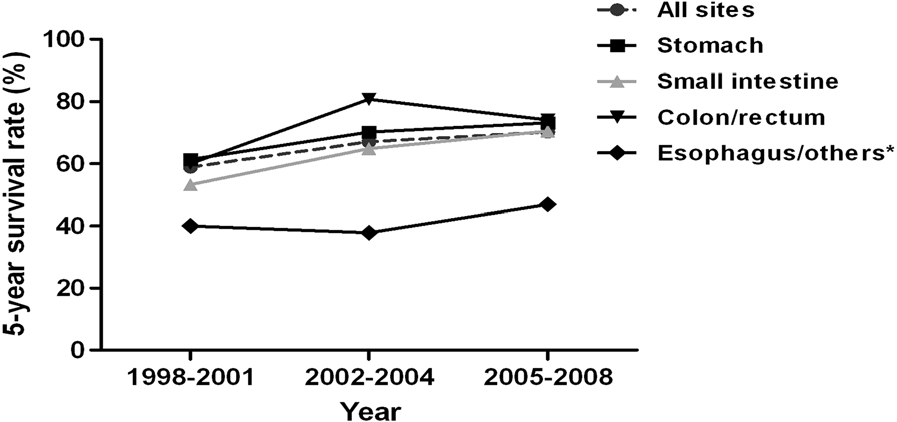
The 5-yr observed overall survival rate of gastrointestinal stromal tumors by primary sites and diagnostic periods (1998–2001, 2002–2004, and 2005–2008), Taiwan, 1998–2008 (^*^Others: retroperitoneum and unspecific sites).

Overall, the 1-yr observed OS rate was 88% and the 5-yr observed OS rate was 66.5% (Table 4). There was a significant sex difference in survival (*p* < .0001) (Table 4). For men, the 1- and 5-yr observed OS rates were 86% and 60.3%, respectively. For women, the 1- and 5-yr observed OS rates were 90.6% and 74.2%, respectively. Patients with GISTs from colon/rectum had the best prognosis with a 5-yr observed OS rate of 72.4%, followed by stomach (69.4%), and small intestine (65.1%) (Table 4). Women had a better prognosis than men for all sites. Among men, the 5-yr observed OS rate was 66.6%, 62.1% and 60.7% for GIST in colon/rectum, stomach, and small intestine, respectively. For women, the 5-yr observed OS rate was 78.7%, 77.5% and 71.3% for GIST in colon/rectum, stomach, and small intestine, respectively. The 5-yr observed OS rate for all patients improved from 58.9% during 1998–2001 to 67.0% during 2002–2004 and to 70.2% during 2005–2008 (Table 4 and Figure 2).

**Table 4:** One and 5-year observed overall survival rates of patients with gastrointestinal stromal tumors, Taiwan, 1998-2008

**Location**	**1998-2008**							
	**1-year survival**				**5-year survival**			
	**Overall**	**Male**	**Female**	***P*** **value**^**a**^	**Overall**	**Male**	**Female**	***P*** **value**^**a**^
All sites	88.0%	86.0%	90.6%	<.0001	66.5%	60.3%	74.2%	<.0001
Stomach	90.1%	87.7%	92.8%	<.0001	69.4%	62.1%	77.5%	<.0001
Small intestine	88.1%	86.5%	90.3%	0.0009	65.1%	60.7%	71.3%	0.0008
Colon/rectum	87.7%	89.5%	85.6%	0.1666	72.4%	66.6%	78.7%	0.0732
Esophagus/others^b^	71.3%	66.4%	77.8%	0.1321	43.4%	36.8%	51.9%	0.0897
	**1998-2001**							
All sites	81.8%	78.5%	86.4%	<.0001	58.9%	52.7%	67.4%	<.0001
Stomach	85.1%	80.4%	90.9%	0.0010	61.4%	53.3%	71.3%	0.0006
Small intestine	77.6%	75.5%	80.9%	0.0336	55.3%	52.5%	59.6%	0.0368
Colon/rectum	80%	80%	80%	0.1118	60%	50%	75%	0.0519
Esophagus/others^b^	60%	75%	--	--	40%	50%	--	--
	**2002-2004**							
All sites	90.0%	87.5%	92.9%	<.0001	67.0%	59.7%	76.0%	<.0001
Stomach	91.0%	88.5%	93.9%	<.0001	70.2%	61.3%	80.1%	<.0001
Small intestine	90.4%	89.3%	92.0%	0.4008	64.9%	59.9%	72.0%	0.2272
Colon/rectum	94.5%	94.9%	94.1%	0.4290	80.8%	74.4%	88.2%	0.3414
Esophagus/others^b^	74.1%	61.3%	88.9%	0.0947	37.9%	29.0%	48.2%	0.1277
	**2005-2008**							
All sites	89.7%	88.6%	90.9%	<.0001	70.2%	65.5%	75.8%	<.0001
Stomach	92.0%	90.9%	93.1%	0.0006	73.2%	68.9%	77.8%	0.0004
Small intestine	91.6%	90.3%	93.4%	0.0081	70.5%	65.9%	76.9%	0.0103
Colon/rectum	86.4%	91.1%	81.4%	0.8154	74.1%	75.3%	73.0%	0.9534
Esophagus/others^b^	70.4%	68.1%	73.6%	0.1976	46.9%	37.7%	58.7%	0.1796

^a^The *P*-value for gender difference was calculated by the Kaplan-Meier method.

^b^Others: retroperitoneum and unspecific sites.

Age, sex, primary site, and the year of diagnosis independently predicted the mortality of GIST (Table 5). Patients older than 50 years had a 1.4 to 5.7 fold increase in the risk of death compared to those younger than 50 years old. Women had a better survival than men (HR = 0.68, 95% CI: 0.60-0.77, *P* < 0.0001). GISTs arising from the stomach had better prognosis than those from the small intestine regardless of the time periods, but this difference became non-statistically significant during 2005–2008 (*P* = 0.12, Additional file 1: Table S1, S2, and S3). Compared with those diagnosed in 2005–2008, patients diagnosed in 1998–2001 (HR = 1.67, 95% CI: 1.42-1.97) and 2002–2004 (HR = 1.25, 95% CI: 1.08-1.46) had a significantly higher risk of mortality (Table 5).

**Table 5:** Survival analysis of patients with gastrointestinal stromal tumors, Taiwan, 1998-2008

	**Univariable**			**Multivariable**		
**Sex**	**HR**^**a**^	**95% CI**^**a**^	***P***	**HR**^**a**^	**95% CI**^**a**^	***P***
Male	Referent			Referent		
Female	0.61	0.54–0.69	<.0001	0.68	0.60–0.77	<.0001
**Age, years**						
< 50	Referent			Referent		
50 to <60	1.23	1.00–1.52	0.053	1.40	1.14–1.73	<.0001
60 to < 70	1.51	1.24–1.83	<.0001	1.74	1.44–2.12	<.0001
70 to < 80	2.73	2.27–3.27	<.0001	3.08	2.56–3.71	<.0001
> 80	4.58	3.64–5.75	<.0001	5.69	4.51–7.17	<.0001
**Primary site**						
Stomach	Referent			Referent		
Small intestine	1.27	1.11–1.44	0.0003	1.35	1.18–1.54	<.0001
Colon/rectum	1.05	0.82–1.34	0.6954	1.08	0.85–1.38	0.5164
Esophagus/others^b^	2.65	2.14–3.27	<.0001	2.51	2.01–3.12	<.0001
**Year of diagnosis**						
2005–2008	Referent			Referent		
1998–2001	1.42	1.21–1.65	<.0001	1.67	1.42–1.97	<.0001
2002–2004	1.23	1.06–1.43	0.0056	1.25	1.08–1.46	0.0035

^a^Hazard ratio (HR) and 95% confidence interval (CI) were calculated based on Cox proportional hazards model.

^b^Others: retroperitoneum and unspecific sites.

## Discussion

Results of this first Asian nation-wide cancer registry-based study of GISTs showed that the annual incidence of GISTs in Taiwan ranged from 1 to 2 cases per 100,000. In a hospital-based retrospective cohort study, Tzen *et al.* estimated that the incidence of GIST in Taiwan during 1998–2004 was 1.37 cases per 100,000, which was similar to our result [18]. In a hospital-based retrospective cohort study, the annual incidence of GISTs in Hong Kong was estimated to be 1.68-1.96 per 100,000 [4]. In a study based on pathology reports from 38 hospitals, Cho *et al*. reported that the incidence of GISTs in Korea was approximately 1.6-2.2 per 100,000 [19]. Studies from Europe and North America reported a GIST incidence of 1.45 per 100,000 in Sweden [20], 0.65-0.90 per 100,000 in Spain [21], 0.6-1.9 per 100,000 in Norway [22], 0.66 in Italy [23], 0.85-1.00 per 100,000 in France [24], 0.9 per 100,000 in Canada [25], 1.32 per 100,000 in United Kingdom [26], and 0.7 per 100,000 in USA [17]. Given the different study time periods and the lack of confirmation by KIT immunohistochemical staining in some studies, it is difficult to compare the incidence rates of GISTs across different countries; however, the published literature to date showed that the incidence rates of GISTs in different countries appeared to fall in a similar range.

Our analysis indicated that the incidence of GISTs in Taiwan increased during 1998–2008, with a more prominent rise since 2002 (Table 3 and Figure 1A). The possible reasons for the observed rise in the incidence of GIST include the improved quality of cancer registration, the advancement of diagnostic technology/method, and the increased awareness of GISTs by physicians which could be partly attributed to the emergence of effective targeted therapeutic agent, imatinib. Previously, GISTs might have been misclassified as leiomyosarcoma, leiomyoma or unspecified sarcoma. The exact diagnosis and tumor origin of GISTs were difficult to determine until the discovery of the gain-of-function mutation in the *KIT* oncogene. In Taiwan, the routine use of CD117 or KIT immunohistochemical staining to diagnose GIST began in 2002. Before 2002, the diagnosis of GIST was based on histology with variable use of staining markers. In addition, no unique code indicating “gastrointestinal stromal sarcoma” was available in the ICD-O-FT, which was used by the TCR before 2002. The rising incidence of GISTs in Taiwan might be attributed to the increased utilization of CD117 staining and the increased awareness of GIST by the physicians. Nevertheless, there was still a rising trend of GIST incidence from 2005 to 2008, during which the use of CD117 or KIT immunohistochemical staining had already been widely adopted for the diagnosis of GISTs. Further follow-up is necessary to clarify whether the incidence of GISTs is truly on the rise. In addition, there was a disproportional rise in the incidence of GIST arising from esophagus/others compared to those from colon/rectum, especially during the 2002–2008 period. (Table 3 and Figure 1B). The increase in the incidence of GIST from esophagus/others resulted mostly from the elevated incidence of GIST located in retroperitoneum and unspecific sites (separate data not shown). The increased awareness of physicians with a more active approach to tumors arising from non-gastrointestinal sites due to the progress in the diagnostic tools and the availability of targeted therapy may partially account for this finding.

In our study, there was a slight male predominance (M/F ratio = 1.0 ~ 1.3) in the incidence of GISTs, which was also observed by studies from Korea (M/F ratio = 1.1) [19], Norway (M/F ratio = 1.6) [22], and the United States (M/F ratio = 1.46) [17]. However, other studies reported a female excess in the number of GISTs [23-26], while one study reported no difference by sex [20]. Taken together, it is not clear whether there is a sex difference in the incidence of GIST, and if existed, may be insignificant. The age distribution of GIST patients in our study is consistent with those reported in the literature, with the majority of GIST patients being diagnosed during the fifth to the seventh decade of life. GISTs are occasionally found in young adults, but rarely among those younger than 18 years of age. In our series, stomach was the most frequent site of involvement (47-59%) followed by small intestine (31-38%) and colon/rectum (6-9%). The site distribution of GISTs in our study is consistent with those published in the previous literature (stomach: 50-64%, small intestine: 17-44%, and colon/rectum: 2-19%) [17,19-26]. In our study, the percentage of GISTs originated from stomach increased with age, while the percentage of GISTs originated in the small intestine decreased with age. To our knowledge, our study is the first to report this interesting finding, which could partially be explained by the more aggressive clinical behavior of small intestine GIST [27]. The more aggressive clinical course and thus the earlier signs and symptoms of GIST from the small intestine as opposed to the more indolent behavior of GIST from other sites may lead to the diagnosis of small intestine GIST at a younger age. However, more investigations are needed to determine the causes for the differences in the percentages of GIST location with increasing age.

Surgery remains the optimal therapy for the curative treatment of GISTs, but unfortunately, more than 50% of patients will develop recurrence or metastasis. Single or combined cytotoxic chemotherapy have failed to yield a satisfactory response. Prior to the introduction of tyrosine kinase inhibitors, the outcome for patients with metastatic disease was poor with a median survival of < 2 years [28]. The prognosis of GIST improved dramatically after the introduction of imatinib, a tyrosine kinase inhibitor approved by the FDA in 2002 for treating KIT-positive GIST [29]. In Taiwan, imatinib became widely prescribed for recurrent or metastatic GISTs, after the approved coverage by the National Health Insurance Administration in 2004. In our analysis by the three time periods, the 5-yr observed OS rate of GISTs improved with the introduction of imatinib as a GIST treatment (1998–2001: 59% vs. 2002–2004: 67%), and further improved with the approved coverage of imatinib by the National Health Insurance Administration (as a proxy for a wider usage) (2005–2008: 70%). This is consistent with previous literature, with GISTs diagnosed in the pre-imatinib era having a 5-year survival ranging from 45% to 63% [4,17,21,22] and GISTs occurring in the imatinib era having a better 5-year survival (79%) [30].

In our analysis, besides the year of diagnosis, female sex, younger age, and stomach location were independent favorable prognostic factors of survival. The impact of the anatomic sites of GIST on survival is equivocal in the literature. In some studies, GIST arising from the stomach was less aggressive than those from other sites while other studies showed no difference [10,31,32]. Our study showed that GIST arising from the stomach had a better survival rate than those affecting the small intestine. Notably, GIST from the colon/rectum exhibited the best 5-yr observed OS of 72.4%, although this survival advantage over GIST in the small intestine disappeared in the multivariable analysis, after adjusting for sex, age, and the year of diagnosis. The difference in survival between GISTs in the stomach and GISTs in the small intestine decreased with time (6.1% in 1998–2001; 5.2% in 2002–2004; 2.7% in 2005–2008), which could be attributed to the advancement in treatment, such as the use of imatinib. In our multivariable analysis, female sex was an independent favorable prognostic factor for survival (Table 5). The magnitude of survival advantage of women over men persisted (Additional file 1: Table S1, S2, S3) even during the era of imatinib treatment. Using SEER data, Tran *et al*. observed a survival advantage of women over men (women vs. men: 5-yr mortality risk HR = 0.83, 95% CI: 0.71-0.97) [17]. Similarly, in another cancer registry-based study of 46 c-KIT confirmed cases diagnosed in 1994–2001, women had a better 5-year survival than men (75% vs. 52%) [21]. In a cohort of 1,215 GISTs patients diagnosed between May, 2000 and October 2010, Call *et al*. also reported a better GIST survival in women compared to men (men vs. women: HR = 1.5, 95% CI: 1.2-1.8) [30]. It is unclear what contributes to the better survival of GISTs among women compared to men and further investigations are warranted.

This study has several strengths. This is the first nation-wide cancer registry-based study of GIST and one of the largest GIST studies from Asia. Because the GIST cases were identified from a nation-wide cancer registry, our results are population-based with a reduced probability of selection bias associated with identifying GISTs from a single or a few medical institutions. The other major strength is the long study period from 1998–2008, which spanned across the eras of pre-imatinib, transition, and imatinib, and allowed us to demonstrate the influence of change in treatment practice on the survival of GIST patients.

This study has several limitations. The TCR does not have complete information on the tumor size of GISTs and lacks data on mitotic index; therefore, risk stratification according to the Armed Forces Institute of Pathology (AFIP) criteria (also known as Miettinen’s criteria) to predict the prognosis of GISTs was not possible [33]. We used multiple ICD-O codes to represent GIST diagnosed in 1998–2001 due to the lack of an ICD-O code specific for GIST and the absence of confirmation by c-KIT staining. As a result, the incidence rates for 1998–2001 might have been overestimated due to the potential inclusion of other non-GIST mesenchymal tumors. However, studies suggested that that the majority of gastrointestinal tumors previously classified as tumors of smooth muscle, including leiomyosarcoma or nerve sheath tumors were GISTs [2,34], which is consistent with our GISTs cases identified for the 1998–2001 period (83.5% was leiomyosarcoma, followed by 8.85% of sarcoma, not otherwise specified, and 3.36% of epithelioid leiomyosarcoma). In addition, compared to GISTs diagnosed during 2002–2008 after the establishment of c-KIT staining as part of the diagnostic protocol and identified by a single ICD-O-3 code (8936: gastrointestinal stromal sarcoma), GISTs from 1998–2001 showed similar distributions of sex, age, and primary sites (Table 1), supporting that the majority of our cases from 1998–2001 were likely GIST. Finally, although our analysis suggested that the introduction and the wider use of imatinib could contribute to the improved survival of GIST patients, it is possible that other factors may have enhanced the survival of GIST patients, including increased awareness of the disease, earlier diagnosis, improved treatment, and better overall population health.

## Conclusions

The incidence of GISTs in Taiwan is comparable to those reported by the US and European studies. GIST is a rare cancer in Taiwan and its incidence has been increasing gradually, partially due to the advancement of diagnostic technology/method and the increased awareness of GISTs by physicians. The occurrence of GISTs is more common in men and the older population. The stomach is the most common primary site followed by the small intestine. Prognostic factors for a better survival of GIST include female sex, younger age, stomach location, and diagnostic years (likely as a proxy for change in treatment practice). Finally, our results suggest that the survival of patients with GIST has improved significantly by targeted therapy.

## Competing interests

The authors declare no conflicts of interest.

## Authors’ contributions

All authors designed the study. CRT and JSC performed statistical analyses. All authors interpreted the results. NJC and JSC drafted the manuscript. All authors read and approved the final manuscript.

## Pre-publication history

The pre-publication history for this paper can be accessed here:

http://www.biomedcentral.com/1471-2407/14/102/prepub

## Supplementary Material

Additional file 1: Table S1. Survival analysis of patients with gastrointestinal stromal tumors, Taiwan, 1998-2001. **Table S2.** Survival analysis of patients with gastrointestinal stromal tumors, Taiwan, 2002-2004. **Table S3.** Survival analysis of patients with gastrointestinal stromal tumors, Taiwan, 2005-2008. Click here for file
