# Supplementary material for: The epidemiology of gastrointestinal stromal tumors in Taiwan, 1998–2008: a nation-wide cancer registry-based study
Source: BMC Cancer. 2014 Feb 18;14:102. doi: 10.1186/1471-2407-14-102 (PMC3932802; doi:10.1186/1471-2407-14-102)
Supplement: Additional file 1: Table S1. — Survival analysis of patients with gastrointestinal stromal tumors, Taiwan, 1998-2001. Table S2. Survival analysis of patients with gastrointestinal stromal tumors, Taiwan, 2002-2004. Table S3. Survival analysis of patients with gastrointestinal stromal tumors, Taiwan, 2005-2008. [file 1471-2407-14-102-S1.doc]

Supplementary Table 1. Survival analysis of patients with gastrointestinal stromal tumors, Taiwan, 1998-2001

|  | Univariable | | | Multivariable | | |
| --- | --- | --- | --- | --- | --- | --- |
| **Sex** | HR^*^ | 95% CI^*^ | *P* | HR^*^ | 95% CI^*^ | *P* |
| Male | Referent |  |  | Referent |  |  |
| Female | 0.62 | 0.50-0.76 | <.0001 | 0.68 | 0.55-0.84 | 0.0005 |
| **Age , years** |  |  |  |  |  |  |
| < 50 | Referent |  |  | Referent |  |  |
| 50 to <60 | 1.33 | 0.95-1.87 | 0.10 | 1.49 | 1.06-2.09 | 0.02 |
| 60 to < 70 | 1.47 | 1.08-1.99 | 0.01 | 1.69 | 1.24-2.29 | 0.0009 |
| 70 to < 80 | 2.24 | 1.66-3.01 | <.0001 | 2.73 | 2.01-3.71 | <.0001 |
| > 80 | 4.27 | 2.79-6.54 | <.0001 | 5.30 | 3.44-8.19 | <.0001 |
| **Primary site** |  |  |  |  |  |  |
| Stomach | Referent |  |  | Referent |  |  |
| Small intestine | 1.47 | 1.18-1.82 | 0.0005 | 1.44 | 1.15-1.80 | 0.001 |
| Colon/rectum | 1.33 | 0.91-1.95 | 0.14 | 1.32 | 0.90-1.95 | 0.15 |
| Esophagus/others^#^ | 1.88 | 0.60-5.87 | 0.28 | 1.18 | 0.38-3.73 | 0.77 |

^*^Hazard ratio (HR) and 95% confidence interval (CI) were calculated based on Cox proportional hazards model.

^#^Others: retroperitoneum and unspecific sites.

Supplementary Table 2. Survival analysis of patients with gastrointestinal stromal tumors, Taiwan, 2002-2004

|  | Univariable | | | Multivariable | | |
| --- | --- | --- | --- | --- | --- | --- |
| **Sex** | HR^*^ | 95% CI^*^ | *P* | HR^*^ | 95% CI^*^ | *P* |
| Male | Referent |  |  | Referent |  |  |
| Female | 0.62 | 0.50-0.76 | <.0001 | 0.66 | 0.53-0.81 | <.0001 |
| **Age , years** |  |  |  |  |  |  |
| < 50 | Referent |  |  | Referent |  |  |
| 50 to <60 | 1.38 | 0.97-1.96 | 0.08 | 1.50 | 1.05-2.14 | 0.03 |
| 60 to < 70 | 1.55 | 1.11-2.16 | 0.01 | 1.75 | 1.25-2.45 | 0.001 |
| 70 to < 80 | 3.02 | 2.21-4.14 | <.0001 | 3.22 | 2.35-4.42 | <.0001 |
| > 80 | 4.81 | 3.21-7.21 | <.0001 | 5.41 | 3.59-8.16 | <.0001 |
| **Primary site** |  |  |  |  |  |  |
| Stomach | Referent |  |  | Referent |  |  |
| Small intestine | 1.24 | 0.99-1.55 | 0.06 | 1.38 | 1.10-1.73 | 0.006 |
| Colon/rectum | 0.72 | 0.47-1.11 | 0.14 | 0.83 | 0.52-1.27 | 0.37 |
| Esophagus/others^#^ | 2.81 | 2.01-3.94 | <.0001 | 3.17 | 2.25-4.46 | <.0001 |

^*^Hazard ratio (HR) and 95% confidence interval (CI) were calculated based on Cox proportional hazards model.

^#^Others: retroperitoneum and unspecific sites.

Supplementary Table 3. Survival analysis of patients with gastrointestinal stromal tumors, Taiwan, 2005-2008

|  | Univariable | | | Multivariable | | |
| --- | --- | --- | --- | --- | --- | --- |
| **Sex** | HR^*^ | 95% CI^*^ | *P* | HR^*^ | 95% CI^*^ | *P* |
| Male | Referent |  |  | Referent |  |  |
| Female | 0.61 | 0.50-0.76 | <.0001 | 0.67 | 0.54-0.83 | 0.0003 |
| **Age , years** |  |  |  |  |  |  |
| < 50 | Referent |  |  | Referent |  |  |
| 50 to <60 | 1.18 | 0.78-1.79 | 0.43 | 1.28 | 0.84-1.94 | 0.25 |
| 60 to < 70 | 1.69 | 1.15-2.48 | 0.008 | 0.88 | 1.28-2.76 | 0.001 |
| 70 to < 80 | 3.36 | 2.34-7.83 | <.0001 | 3.65 | 2.54-5.25 | <.0001 |
| > 80 | 5.65 | 3.78-8.45 | <.0001 | 6.77 | 4.50-10.20 | <.0001 |
| **Primary site** |  |  |  |  |  |  |
| Stomach | Referent |  |  | Referent |  |  |
| Small intestine | 1.11 | 0.88-1.41 | 0.37 | 1.21 | 0.95-1.53 | 0.12 |
| Colon/rectum | 1.17 | 0.75-1.82 | 0.50 | 1.12 | 0.72-1.76 | 0.61 |
| Esophagus/others^#^ | 2.93 | 2.18-3.95 | <.0001 | 2.19 | 1.60-3.00 | <.0001 |

^*^Hazard ratio (HR) and 95% confidence interval (CI) were calculated based on Cox proportional hazards model.

^#^Others: retroperitoneum and unspecific sites.
